# Recent advances in unlinked total elbow arthroplasty in Japan

**DOI:** 10.1016/j.jseint.2023.10.005

**Published:** 2023-11-28

**Authors:** Takuji Iwamoto, Hiroyasu Ikegami, Atsushi Tanji, Yasuhiro Kiyota, Taku Suzuki, Noboru Matsumura, Kazuki Sato

**Affiliations:** a Department of Orthopedic Surgery, Keio University School of Medicine, Tokyo, Japan; b Department of Orthopedic Surgery, Toho University Ohashi Medical Center, Tokyo, Japan; c Department of Orthopedic Surgery, Japanese Red Cross Ashikaga Hospital, Ashikaga, Japan; d Institute for Integrated Sports Medicine, Keio University School of Medicine, Tokyo, Japan

**Keywords:** Total elbow arthroplasty, Unlinked type, Surgical planning, Surgical approach, Implants, Augmented reality–assisted surgery

## Abstract

**Background:**

Total elbow arthroplasty (TEA) is a valuable therapeutic approach for improving function and relieving pain in severely deformed elbow joints. However, TEA is associated with a high incidence of complications. In Japan, the use of unlinked TEA has a long history, with the development of the Kudo prosthesis marking a significant milestone. Subsequently, various unlinked implant designs have been developed. Although favorable long-term clinical results have been reported, complications remain a concern. To further improve the outcome of unlinked TEA, attempts have been made in recent years to develop various surgical approaches and intraoperative support devices. This review focuses on the clinical outcomes and recent advances in unlinked TEA in Japan.

**Methods:**

A comprehensive review of clinical results and advancements in unlinked TEA in Japan was conducted. The analysis included trends in the number of TEA, medium-term and long-term results for unlinked TEA, surgical approaches, or preoperative planning techniques.

**Results:**

Several implant designs have been developed in Japan. Clinical studies have reported satisfactory long-term outcomes with these implants, but complications, such as infection, fractures, and dislocation, have been observed. In order to enhance the outcomes of unlinked TEA, various triceps-on approaches have been developed as alternatives to the triceps-detaching approach, which compromises the continuity between the triceps tendon and ulna, leading to inevitable complications related to the triceps tendon. Preservation or repair of the surrounding soft tissues is considered critical for preventing postoperative instability due to the absence of a radial head in the current unlinked TEA design. Computed tomography-based 3-dimensional preoperative planning has been pioneered in Japan, demonstrating its effectiveness in predicting implant size and achieving appropriate implant placement. Additionally, augmented reality–assisted surgery is being explored to accurately translate preoperative planning into the surgical procedure.

**Conclusion:**

Unlinked TEA for inflammatory arthritis has exhibited promising long-term results in Japan, with ongoing efforts to improve surgical techniques and preoperative planning. Further advancements are anticipated to prevent complications such as dislocation and peri-implant fractures.

Total elbow arthroplasty (TEA) is a useful therapeutic approach for reconstructing the elbow joint for improved function, providing pain relief and improved range of motion in severely deformed elbow joints. Although TEA is primarily used to treat rheumatoid arthritis (RA), it is also used to treat osteoarthritis and post-traumatic arthritis. Total elbow arthroplasty (TEA) has recently gained attention as a 1-stage reconstruction method for severely comminuted distal humerus fractures.^5^ Although TEA offers favorable functional recovery, it is associated with a high incidence of complications, with a reported overall complication rate of 24.3% based on a systematic review.^31^ In Japan, the use of unlinked TEA dates back several decades, beginning with the development of the Kudo elbow prosthesis. To reduce postoperative dislocation and instability, which are major complications associated with unlinked TEA, improvements in implants’ design, surgical approaches, and intraoperative support devices have been attempted. This review focuses on the clinical results and recent advances of unlinked TEA in Japan. We conducted a literature search using the PubMed database, with search criteria including the term "total elbow" in the title and affiliation from Japan. The inclusion criteria were as follows: publication within the last decade, English as the publication language, and analysis covering trends in the number of TEA, medium-term and long-term results for unlinked TEA, surgical approaches, or preoperative planning techniques. As a result, we have cited 9 of them in this review.

## Trends in the number of TEA

Total elbow arthroplasties (TEAs), compared with hip and knee replacements, are less frequently performed, as RA is the primary target disease. Additionally, the Swedish registry shows a downward trend in the use of TEAs for RA, as orthopedic surgery for RA has been declining with recent advances in pharmacotherapy.^32^ On the other hand, the number of TEA procedures for traumatic injuries has shown an increasing trend in recent years.^9^ The United States database shows that the number of TEA procedures has doubled from 0.45/100,000 in 1998 to 0.96/100,000 in 2011.^26^ The German database also shows an 84% increase in the number of TEAs from 2005 to 2014, with an increase in indications for trauma from 12% to 42%, along with a 2-fold increase in the indication for revision cases.^13^ In Japan, there hasn't been accurate reporting on the trend of the number of TEAs due to the absence of a large-scale database. However, in 2020, the Japanese Orthopaedic Association National Registry was launched, and it is expected that future analyses will provide valuable insights into this matter.^29^ In addition, the life expectancy of patients with RA is improving with advances in pharmacotherapy.^33^ Therefore, treatment plans should consider possible revision surgeries that may be needed in the future.

## Implants for TEA

Implants used for TEA are classified into 2 types: the linked type, in which the humeral and ulnar implants are mechanically linked, and the unlinked type, in which the implants are not linked. The linked type is widely used worldwide because it has the advantage of not causing dislocation, a serious postoperative complication. However, its disadvantage is that the long stem of the implant must be cemented into the medullary cavity, making reoperation difficult.^8^ On the other hand, the unlinked type reconstructs the elbow joint in a shape that is anatomically similar to an elbow joint and reduces the constraint between implants, allowing for cementless placement. Theoretically, unlinked-type implants may be less prone to loosening, but postoperative dislocation and instability may occur. In cases where the unlinked type is indicated, an extent of bone mass and soft tissue balance must be maintained. The unlinked type is difficult to apply in rheumatoid elbow joints with severe bone resorption or in traumatic cases.

One factor that causes postoperative instability in the unlinked type is radial head resection. The majority of unlinked TEAs lack a radial head prosthesis, with only a few being designed with it.^7^^,^^22^ Although in theory, the radial head component improves soft tissue balance and has been shown to improve stability in in vitro biomechanical studies,^10^ in vivo results with radial head components have not been favorable. The placement of a radial head implant in the proper position when reconstructing a proximal radio-ulnar joint is difficult, and the placement of the radial head poses the risks of polyethylene wear and implant loosening.^24^ In recent years, convertible type implants, which can be used intraoperatively as linked or unlinked, have been developed.^27^ Although convertible type implants have the advantage of being able to be used as a linked-type implant without it being removed when postoperative dislocations occur, the design of the implant is similar to that of a linked-type implant rather than an unlinked-type implant.

## Development of unlinked type TEA and surgical results in Japan

**Figure 1:**
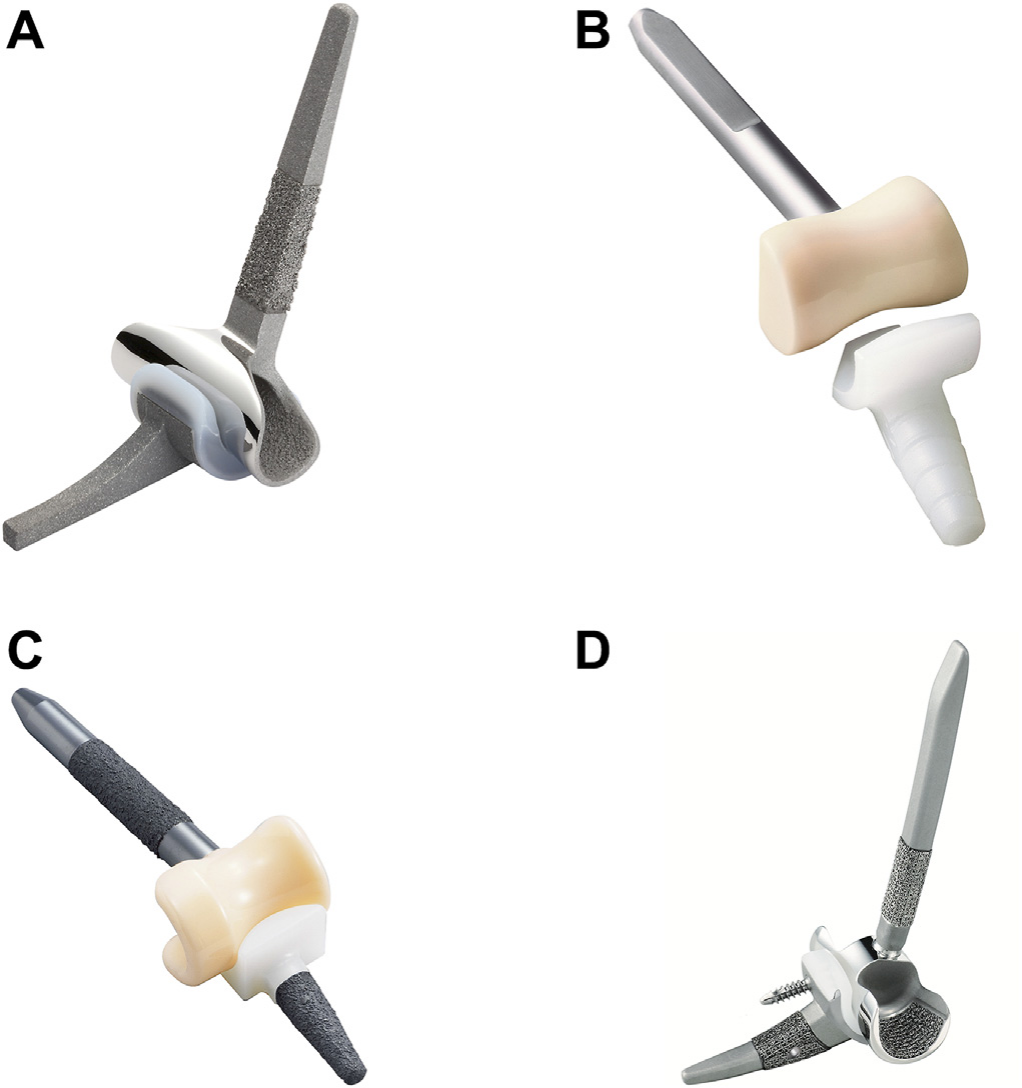
Unlinked TEA development and its use in Japan. (**A**) K-Elbow (Zimmer Biomet G.K., Warsaw, IN, USA) (**B**). JACE (KYOCERA Co., Kyoto, Japan) (**C**). MNSK (KYOCERA Co., Kyoto, Japan) (**D**). K-NOW (Teijin-Nakashima Medical Co., Ltd., Okayama, Japan). These photos are provided with permission from the respective companies. *TEA*, total elbow arthroplasty.

**Figure 2:**
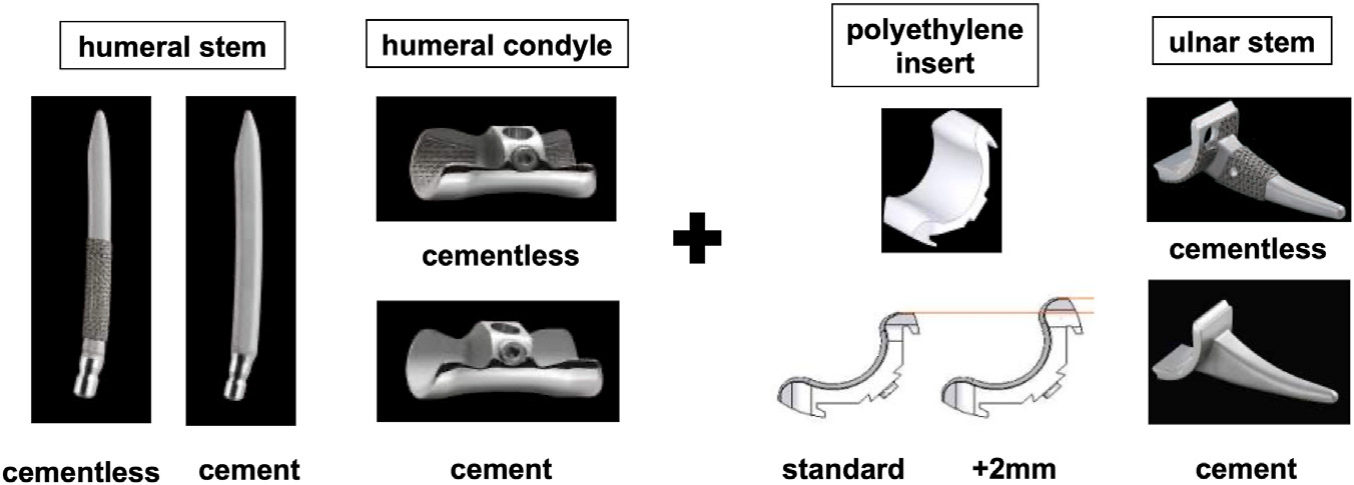
The system of K-NOW (Teijin-Nakashima Medical Co., Ltd., Okayama, Japan) unlinked TEA. The humeral and ulnar stems can be cementless or cemented. In addition, K-NOW allows the surgeon to control posterior instability by selecting a deeper covering for the polyethylene insert. *TEA*, Total elbow arthroplasty.

The original Kudo prosthesis was introduced in 1972.^18^ The initial design of both the Type 1 and Type 2 prostheses had unstemmed humeral components with a cylindrical articulation; however, the humeral component was redesigned as a stemmed component owing to the high incidence of early loosening.^17^ Humeral articulation has a saddle design that allows for medial-lateral translation of the ulnar component. Based on this design, the Kudo elbow prosthesis has undergone continuous improvements, and a Type 6 K-Elbow (Zimmer Biomet, Warsaw, IN, USA), consisting of a Cobalt Chromium alloy humeral component and a metal-backed polyethylene ulnar component, is currently in use. Since the development of the Kudo prosthesis, a number of unlinked-type implants, including JACE (KYOCERA Co., Kyoto, Japan)^19^ and MNSK (KYOCERA Co., Kyoto, Japan)^16^ that have an alumina ceramic sliding surface and K-NOW (Teijin-Nakashima Medical Co., Ltd., Okayama, Japan)^11^ a modular type that can separate the humeral stem and condyle, have been developed in Japan and are currently being used clinically (Fig. 1). K-NOW also allows the surgeon to select a deeper covering for the polyethylene insert and control posterior instability (Fig. 2).

Table I summarizes papers on medium-term and long-term results of unlinked TEA in Japan. Kodama et al reported the long-term results of unlinked TEA in Japan, with an average observation period of 141 months in 41 patients with Kudo type 5 elbow prosthesis. They reported a good 5-year survival rate of 87.8%, but a 10-year survival rate of 70.7%, indicating the occurrence of ulnar component loosening.^15^ They also reported that, during Kudo type 5 elbow prosthesis revision surgery, the neck of the all-polyethylene ulnar component was fractured in 3/9 cases.^14^ The current Kudo type 6 ulnar component has a metal back and is expected to improve long-term results. Nishida et al reported the mid-term clinical results of cemented unlinked JACE, with an average observation period of 9 years in 87 elbows, showing an excellent 93% survival rate up to 14 years with any revision surgery as the endpoint.^19^ They also reported the long-term outcomes of unlinked TEA for RA in patients younger than 50 years old. During a mean follow-up period of 13.6 years, there were no revisions of implant removal and no radiological evidence of loosening around the components; they reported a 25-year survival rate of 78.1% with any revision surgery as the endpoint.^20^ Kondo et al reported favorable long-term clinical results of the MNSK system in 75 patients, with a 5-year postoperative survival rate of 97% and a 10-year survival rate of 93%. While good long-term results have been achieved, complications are not uncommon. Complications were observed in 9/75 elbows (12%) and included periprosthetic infection (3 elbows), fracture (4 elbows), and dislocation (2 elbows).^16^ These results suggest that unlinked TEA can provide good long-term results if acute complications can be avoided. To further improve the outcome of unlinked TEA, recent attempts have been made to devise new surgical approaches and 3-dimensional (3D) preoperative planning.

**Table I:** Medium-term and long-term results of unlinked TEA in Japan.

Author	Prosthesis	Cement/Cementless	No. of patient	Average age at surgery	Larsen grade	Follow-up period	Survival rate (any revision surgery)	Complications		
								Infection	Periprosthetic fracture	Dislocation
Kodama et al^15^	Kudo type5	humerus cementless/ulna cemented	41 elbows (31 patients)	58.9	G4:21, G5:20	11.8 y (10-16.9)	5 y: 90.2% 10 y: 75.6%	1		
Nishida et al^19^	JACE	humerus cemented/ulna cemented	87 elbows (75 patients)	61.8	G3:4, G4:78, G5:5	8.9 y (2-14)	10 y: 93%	1	4	1
Kondo et al^16^	MNSK	humerus cementless/ulna cemented	75 elbows (67 patients)	64	G3:5, G4:67, G5:3	5.2 y (2-11)	5 y: 97% 10 y: 93%	3	4	2

*G*, grade; *y*, year.

## Surgical approaches for TEA

Various methods for the posterior approach in TEA for the elbow joint, which can be broadly classified into the triceps-detaching (triceps-off) and triceps-sparing (triceps-on) approaches, have been reported. The triceps-detaching (triceps-off) approach is generally being used in TEA because of the need to secure a sufficient surgical field. Common posterior approaches include the Campbell approach,^4^ which preserves the olecranon attachment of the triceps tendon and requires a V-shaped incision to be made and reversed distally, and the Bryan–Morrey approach,^3^ which ensures that the triceps tendon is continuous to the ulna periosteum but is completely detached from the olecranon. Both methods compromise the continuity between the triceps tendon and ulna, and complications related to the triceps tendon are inevitable. The triceps tendon is important as a dynamic stabilizer of the elbow joint, and maintaining the continuity of the triceps tendon is essential to prevent postoperative instability after unlinked TEA.^25^

**Figure 3:**
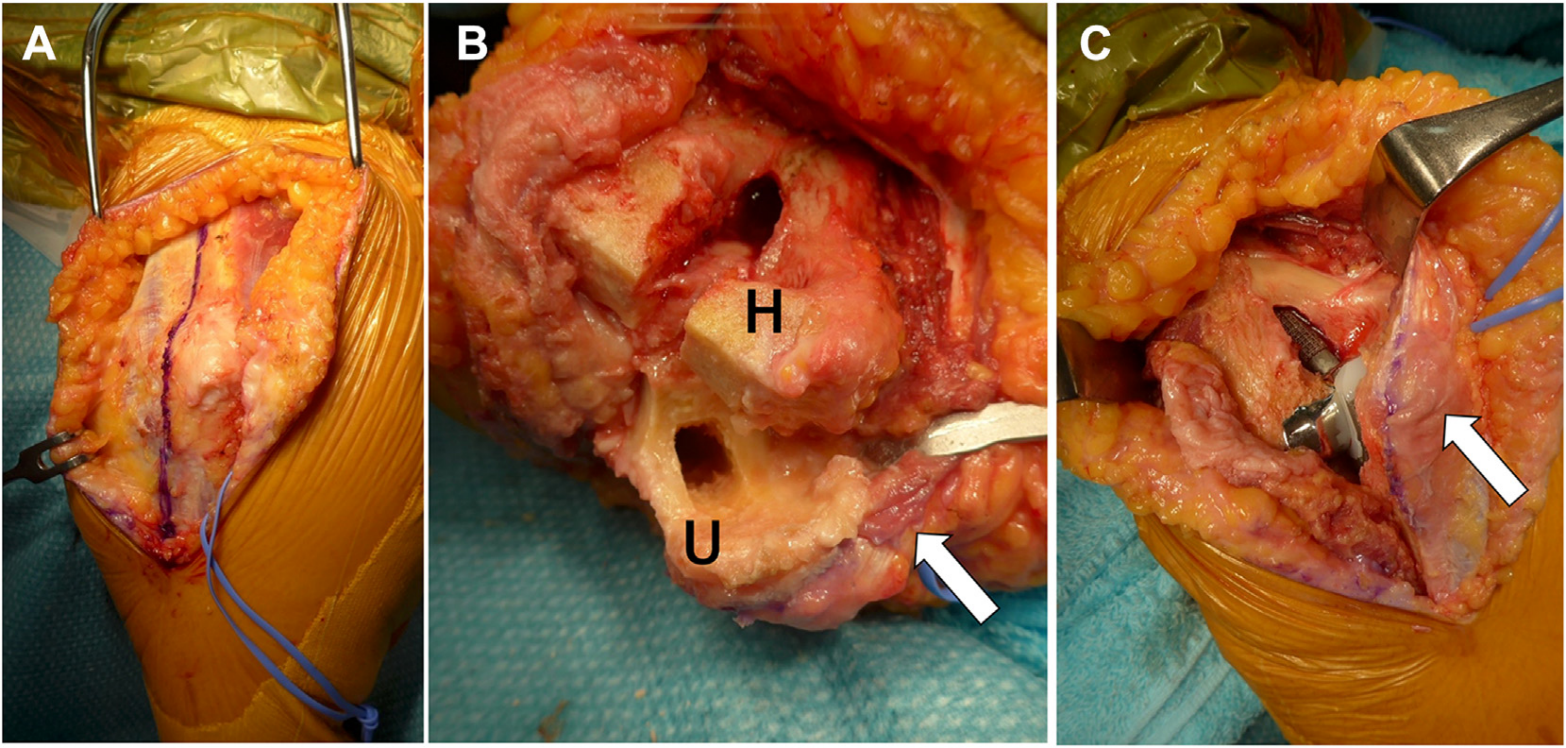
Intraoperative photograph of unlinked TEA using the lateral paraolecranon approach. (**A**) The triceps brachii is divided longitudinally in the midline, and the anchoneus muscle is detached from the ulna at the lateral margin of the olecranon. (**B**) After resecting the radial head, the elbow joint is exposed from the lateral side, and osteotomy is performed while ensuring continuity of the triceps brachii (arrow). (**C**) After placement of the implants. *TEA*, total elbow arthroplasty; *H*, humerus; *U*, ulna.

On the other hand, the triceps-on approach, which maintains continuity between the triceps tendon and ulna bone, has also been attempted in TEA. The bilaterotricipital approach,^1^ which requires that both sides of the triceps muscle is approached, provides a better range of motion compared with that observed with the triceps-off approach. Furthermore, it was previously reported that triceps tendon rupture occurred in 15.2% of patients who underwent the triceps-off approach, whereas none occurred with the bilaterotricipital approach.^6^ However, reports on the use of the bilateral triceps approach for TEA have been few because of limited surgical exposure. Studer et al^28^ reported a lateral paraolecranon approach, which requires that the triceps brachii is divided longitudinally in the midline and the anchoneus muscle is detached from the ulna at the lateral margin of the olecranon. After resecting the radial head, the elbow joint can be exposed from the lateral side without difficulty; our facility uses this approach during unlinked TEA (Fig. 3).^12^ Oizumi et al from Japan reported on the triceps-sparing approach, which requires that the elbow joint is approached from the medial side of the triceps brachii without dissection of the triceps tendon.^21^ In this study, linked-type implants were used, but it may also be applicable to unlinked-type implants. On the other hand, Nishida et al reported a technique with a low dislocation rate when used during unlinked TEA with preservation of the medial collateral ligament.^19^ Because the current design for unlinked TEA does not use a radial head, preservation or repair of the soft tissue is considered critical to prevent postoperative instability.

## Surgical planning for TEA

**Figure 4:**
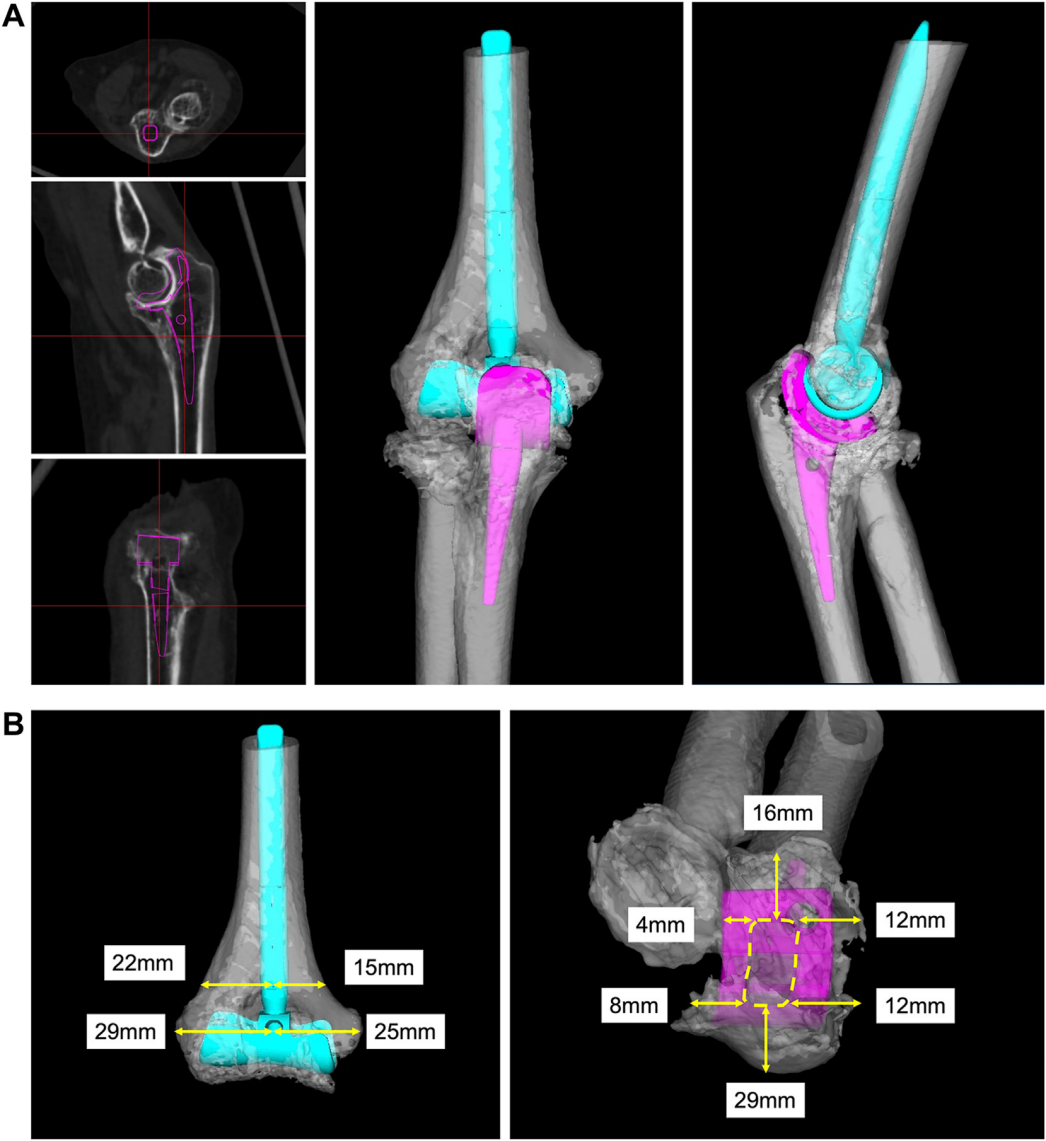
Computed tomograhy-based 3D preoperative planning for TEA. (**A**) The implant size and position are determined to fit the humeral and ulnar canals in the coronal, sagittal, and axial views (Left). Humeral component rotation is positioned parallel to the humeral epicondylar axis. Ulnar component rotation is oriented parallel to the surface of the humeral component. (**B**) To reproduce the planned placement position during surgery, several parameters around the bone tunnel are determined, and these measurements are used as a reference for implant placement during surgery. *3D*, 3-dimensional; *TEA*, total elbow arthroplasty.

In rheumatoid elbows with severe bone destruction, accurate implant placement based on proper preoperative planning is important for preventing complications such as intraoperative fractures and rotational malalignment. Brownhill et al reported in a cadaveric study that the internal-external and varus-valgus malrotations of the linked-type humeral component increased implant loading.^2^ However, it is difficult to obtain accurate antero-posterior and lateral radiographs in cases of advanced joint destruction, making it difficult to accurately perform preoperative planning using conventional 2-dimensional templates.^23^ Although reports on 3D preoperative planning in the knee and hip joints are numerous, those on TEA are few. Computed tomography-based 3D preoperative planning in TEA was first developed in Japan, and its effectiveness in predicting implant size and appropriate implant placement has been reported.^12^ In 3D preoperative planning, the implant size is determined to fit the humeral and ulnar canals in the coronal, sagittal, and axial views. Humeral component rotation is positioned parallel to the humeral epicondylar axis. Ulnar component rotation is oriented parallel to the surface of the humeral component (Fig. 4, *A*). To reproduce the planned placement position during surgery, several parameters around the bone tunnel are determined, and these measurements are used as a reference for implant placement during surgery (Fig. 4, *B*). Augmented reality–assisted surgery for TEA is also being attempted to accurately reflect preoperative planning during surgery.^30^ The development of intraoperative support devices for the elbow joint, which has a complex bone morphology, is expected to reduce complications.

## Conclusion

The number of TEAs is much less than that of lower limb arthroplasties, making it difficult to improve implants and surgical techniques. While the long-term outcomes of unlinked TEA for inflammatory arthritis have improved, further advancements are anticipated to prevent complications such as dislocation and peri-implant fractures.

## Disclaimers:

Funding: No funding was disclosed by the authors.

Conflicts of interest: The authors, their immediate families, and any research foundation with which they are affiliated have not received any financial payments or other benefits from any commercial entity related to the subject of this article.

## References

[1] Alonso-Llames M. (1972). Bilaterotricipital approach to the elbow. Its application in the osteosynthesis of supracondylar fractures of the humerus in children. Acta Orthop Scand.

[2] Brownhill J.R., Pollock J.W., Ferreira L.M., Johnson J.A., King G.J. (2012). The effect of implant malalignment on joint loading in total elbow arthroplasty: an in vitro study. J Shoulder Elbow Surg.

[3] Bryan R.S., Morrey B.F. (1982). Extensive posterior exposure of the elbow. A triceps-sparing approach. Clin Orthop Relat Res.

[4] Campbell W.C. (1922). Arthroplasty of the elbow. Ann Surg.

[5] Cobb T.K., Morrey B.F. (1997). Total elbow arthroplasty as primary treatment for distal humeral fractures in elderly patients. J Bone Joint Surg Am.

[6] Dachs R.P., Fleming M.A., Chivers D.A., Carrara H.R., Du Plessis J.P., Vrettos B.C. (2015). Total elbow arthroplasty: outcomes after triceps-detaching and triceps-sparing approaches. J Shoulder Elbow Surg.

[7] Ewald F.C., Scheinberg R.D., Poss R., Thomas W.H., Scott R.D., Sledge C.B. (1980). Capitellocondylar total elbow arthroplasty. J Bone Joint Surg Am.

[8] Foruria A.M., Sanchez-Sotelo J., Oh L.S., Adams R.A., Morrey B.F. (2011). The surgical treatment of periprosthetic elbow fractures around the ulnar stem following semiconstrained total elbow arthroplasty. J Bone Joint Surg Am.

[9] Gay D.M., Lyman S., Do H., Hotchkiss R.N., Marx R.G., Daluiski A. (2012). Indications and reoperation rates for total elbow arthroplasty: an analysis of trends in New York state. J Bone Joint Surg Am.

[10] Inagaki K., O’Driscoll S.W., Neale P.G., Uchiyama E., Morrey B.F., An K.N. (2002). Importance of a radial head component in Sorbie unlinked total elbow arthroplasty. Clin Orthop Relat Res.

[11] Iwamoto T., Ikegami H., Suzuki T., Oki S., Matsumura N., Nakamura M. (2018). The history and future of unlinked total elbow arthroplasty. Keio J Med.

[12] Iwamoto T., Suzuki T., Oki S., Matsumura N., Nakamura M., Matsumoto M. (2018). Computed tomography–based 3-dimensional preoperative planning for unlinked total elbow arthroplasty. J Shoulder Elbow Surg.

[13] Klug A., Gramlich Y., Buckup J., Schweigkofler U., Hoffmann R., Schmidt-Horlohé K. (2018). Trends in total elbow arthroplasty: a nationwide analysis in Germany from 2005 to 2014. Int Orthop.

[14] Kodama A., Mizuseki T., Adachi N. (2018). Macroscopic investigation of failed Kudo type 5 total elbow arthroplasty. J Shoulder Elbow Surg.

[15] Kodama A., Mizuseki T., Adachi N. (2017). Kudo type-5 total elbow arthroplasty for patients with rheumatoid arthritis: a minimum ten-year follow-up study. Bone Joint J.

[16] Kondo N., Arai K., Fujisawa J., Murai T., Netsu T., Endo N. (2019). Clinical outcome of Niigata-Senami-Kyocera modular unconstrained total elbow arthroplasty for destructive elbow in patients with rheumatoid arthritis. J Shoulder Elbow Surg.

[17] Kudo H., Iwano K. (1990). Total elbow arthroplasty with a non-constrained surface-replacement prosthesis in patients who have rheumatoid arthritis. A long-term follow-up study. J Bone Joint Surg Am.

[18] Kudo H., Iwano K., Watanabe S. (1980). Total replacement of the rheumatoid elbow with a hingeless prosthesis. J Bone Joint Surg Am.

[19] Nishida K., Hashizume K., Nasu Y., Ozawa M., Fujiwara K., Inoue H. (2018). Mid-term results of alumina ceramic unlinked total elbow arthroplasty with cement fixation for patients with rheumatoid arthritis. Bone Joint J.

[20] Nishida K., Nasu Y., Hashizume K., Okita S., Nakahara R., Saito T. (2023). Outcome of unlinked total elbow arthroplasty for rheumatoid arthritis in patients younger than 50 years old. Bone Jt Open.

[21] Oizumi N., Suenaga N., Yoshioka C., Yamane S. (2015). Triceps-sparing ulnar approach for total elbow arthroplasty. Bone Joint J.

[22] Pritchard R.W. (1983). Anatomic surface elbow arthroplasty. A preliminary report. Clin Orthop Relat Res.

[23] Prkić A., van Bergen C.J.A., The B., Eygendaal D. (2016). Pre-operative templating in total elbow arthroplasty: not useful. Arch Orthop Trauma Surg.

[24] van Riet R.P., Morrey B.F., O’Driscoll S.W. (2009). The Pritchard ERS total elbow prosthesis: lessons to be learned from failure. J Shoulder Elbow Surg.

[25] Ring D. (2008). Instability after total elbow arthroplasty. Hand Clin.

[26] Singh J.A., Ramachandran R. (2016). Sex differences in characteristics, utilization, and outcomes of patient undergoing total elbow arthroplasty: a study of the US nationwide inpatient sample. Clin Rheumatol.

[27] Strelzow J.A., Frank T., Chan K., Athwal G.S., Faber K.J., King G.J.W. (2019). Management of rheumatoid arthritis of the elbow with a convertible total elbow arthroplasty. J Shoulder Elbow Surg.

[28] Studer A., Athwal G.S., Macdermid J.C., Faber K.J., King G.J. (2013). The lateral para-olecranon approach for total elbow arthroplasty. J Hand Surg Am.

[29] Taneichi H., Kanemura T., Inoue G., Iwase Y., Ueda H., Kuzuhara A. (2023). Current status and future prospects of the Japanese orthopaedic association national registry (JOANR), Japan's first national registry of orthopaedic surgery. J Orthop Sci.

[30] Tanji A., Nagura T., Iwamoto T., Matsumura N., Nakamura M., Matsumoto M. (2022). Total elbow arthroplasty using an augmented reality–assisted surgical technique. J Shoulder Elbow Surg.

[31] Voloshin I., Schippert D.W., Kakar S., Kaye E.K., Morrey B.F. (2011). Complications of total elbow replacement: a systematic review. J Shoulder Elbow Surg.

[32] Weiss R.J., Ehlin A., Montgomery S.M., Wick M.C., Stark A., Wretenberg P. (2008). Decrease of RA-related orthopaedic surgery of the upper limbs between 1998 and 2004: data from 54579 Swedish RA inpatients. Rheumatology.

[33] Zhang Y., Lu N., Peloquin C., Dubreuil M., Neogi T., Aviña-Zubieta J.A. (2017). Improved survival in rheumatoid arthritis: a general population-based cohort study. Ann Rheum Dis.

